# Pathogens Use and Abuse MicroRNAs to Deceive the Immune System

**DOI:** 10.3390/ijms17040538

**Published:** 2016-04-09

**Authors:** Thomas B. Flór, Bianca Blom

**Affiliations:** Department of Cell Biology and Histology, Academic Medical Center, University of Amsterdam, Amsterdam 1105 AZ, The Netherlands; thomas_flor@hotmail.com

**Keywords:** epigenetics, pathogens, immune system, microRNA

## Abstract

Emerging evidence has demonstrated that microRNAs (miRs) play a role in the survival and amplification of viruses, bacteria and other pathogens. There are various ways in which pathogens can benefit from miR-directed alterations in protein translation and signal transduction. Members of the herpesviridae family have previously been shown to encode multiple miRs, while the production of miRs by viruses like HIV-1 remained controversial. Recently, novel techniques have facilitated the elucidation of true miR targets by establishing miR-argonaute association and the subsequent interactions with their cognate cellular mRNAs. This, in combination with miR reporter assays, has generated physiologically relevant evidence that miRs from the herpesviridae family have the potential to downregulate multiple cellular targets, which are involved in immune activation, cytokine signaling and apoptosis. In addition, viruses and bacteria have also been linked to the induction of host cellular miRs, which have the capacity to mitigate immune activation, cytokine signaling and apoptosis. Interfering with miR expression may be clinically relevant. In the case of hepatitis C infection, the cellular miR-122 is already targeted therapeutically. This not only exemplifies how important miRs can be for the survival of specific viruses, but it also delineates the potential to use miRs as drug targets. In this paper we will review the latest reports on viruses and bacteria that abuse miR regulation for their benefit, which may be of interest in the development of miR-directed therapies.

## 1. Introduction

On a daily basis we are surrounded by billions of pathogens, including viruses and bacteria. Evolution has armed vertebrates with an innate and adaptive immune system, which provides us with elaborate and extensive mechanisms to counteract invasion by such pathogens [1,2]. The war between host and pathogen still wages on, and pathogens use and abuse every host mechanism that will give them the edge in survival and amplification. This includes the regulatory functions of short non-coding RNAs (sncRNA), like microRNAs (miRs).

### 1.1. MicroRNAs (miRs)

The miRs are short, single-stranded, non-coding molecules that are about 18–25 nucleotides long. They are derived from a 65–75 bp pre-miR that is transcribed by an RNA polymerase II and cleaved by Drosha and DiGeorge syndrome critical region gene 8 (DGCR8). Subsequent association with exportin-5 results in translocation of miRs to the cytoplasm, where the complex associates with Dicer, two dimerization domains in the trans-activation response RNA-binding protein (TRBP) and Argonaute (Ago) [3,4]. Here, the Piwi, Argonoute and Zwille (PAZ) domains of Dicer interact with the RNA ends of the stem loop and the RNase III domain cuts off the loop, leaving a double-stranded miR (dsmiR) 18–25 nucleotides long. After generation of the dsmiR, Dicer and TRBP are released and the dsmiR is unwound by a still unspecified helicase. Together, the unwound single-stranded miR and Ago form the so-called RNA-induced silencing complex (RISC). In humans, one of four Argonoute (Ago) paralogs binds the 5′-untranslated region (UTR) of the miR with a pocket near the Mid domain, while the 3′-UTR binds the PAZ domain [5]. Moreover, the RNase H fold in the P-element induced wimpy testis (PIWI) domain of Ago2 can exhibit guide strand-dependent endonucleolytic cleavage. This domain also allows for the binding with glycine (G)-tryptophan(W) containing protein of 182 kDa, which has an important role in the formation of GW/P bodies and translational repression. Perfect miR-mRNA complementarity promotes mRNA degradation by mRNA 3′ deadenylation and decapping by decapping protein-1 (Dcp1/2) and Xrn1p, while imperfect matching is associated with translational repression [6,7,8,9]. Yet, the effects of RISC are not restricted to silencing, since binding to the 5′-end of the mRNA can also increase translation [10,11]. Recently, the development of novel techniques, such as deep sequencing and photoactivatable ribonucleoside-enhanced crosslinking and immunoprecipitation (PAR-CLIP) have allowed for more reliable analysis of miR-Ago association, which is an important indicator that a miR is functionally relevant [5]. During PAR-CLIP, 4-thiouridine (4SG) or 6-thioguanosine (6SG) residues are incorporated into RNA transcripts and crosslinked via 365 nm UV rays to proteins. Subsequent immunoprecipitation and sequence analysis can then be used to identify the exact binding site of RNA binding proteins like Ago [12]. One of the advantages of these methods is that it can detect miR-Ago interactions *in vivo*, which in combination with deep sequencing and reporter assays, can provide clear indications that pathogen-derived miRs can assume a regulatory role in host mRNA translation [13].

### 1.2. Immune Responses

Immune activation coincides with the binding of pathogen-associated molecular patterns (PAMPS) to pathogen recognition receptors (PRRs), including toll-like receptors (TLRs), nucleotide-binding oligomerization domain receptors (NLRs), retinoic acid-inducible gene 1 (RIG-I)-like receptors and C-type lectin receptors. Affinity of RISC for the mRNAs encoding such receptors and/or their signal transducers has been shown to impact transcription and immune activation. In the case of TLRs, this can result in decreased translation of signal transducers like Interleukin-1 receptor-associated kinase 1 (IRAK1) and TNF receptor-associated factor 6 (TRAF6), thereby affecting transcription activation by activator protein-1 (AP-1) and nuclear factor kappa-light-chain-enhancer of activated B cells (NF-κB) [6,14,15,16,17]. In addition, miRs have also been found to target the C-type lectin receptor dendritic cell-specific intercellular adhesion molecule-3-grabbing non-integrin (DC-SIGN) [18,19]. T cell activation and natural killer (NK) cell effector functions can be influenced by miRs regulating phosphatase concentrations, which have a regulatory function in downstream costimulatory and co-inhibitory receptor signaling [20,21]. What is more, miR-induced alterations in the levels of these phosphatases have been shown to affect gene transcription in T cells. Therefore, disruption of signaling downstream of PRRs and T cell receptors (TCRs) can affect the production of cytokines and the level of immune activation. Signals induced by cytokines can also be subjected to miR regulation, which is evidenced by the miR targeting of cytokine receptors, signal transducers and activators of transcription (STATs), suppressors of cytokine signaling (SOCS) and small mothers against decapentaplegic (SMADs) [22,23,24]. Admittedly, evidence of miR regulation is often derived from *in vitro* experiments; however, the effects of alterations in miR quantities and expression patterns do suggest that they have an essential role in fine tuning the immune response *in vivo*.

### 1.3. Scope of the Review

**Figure 1:**
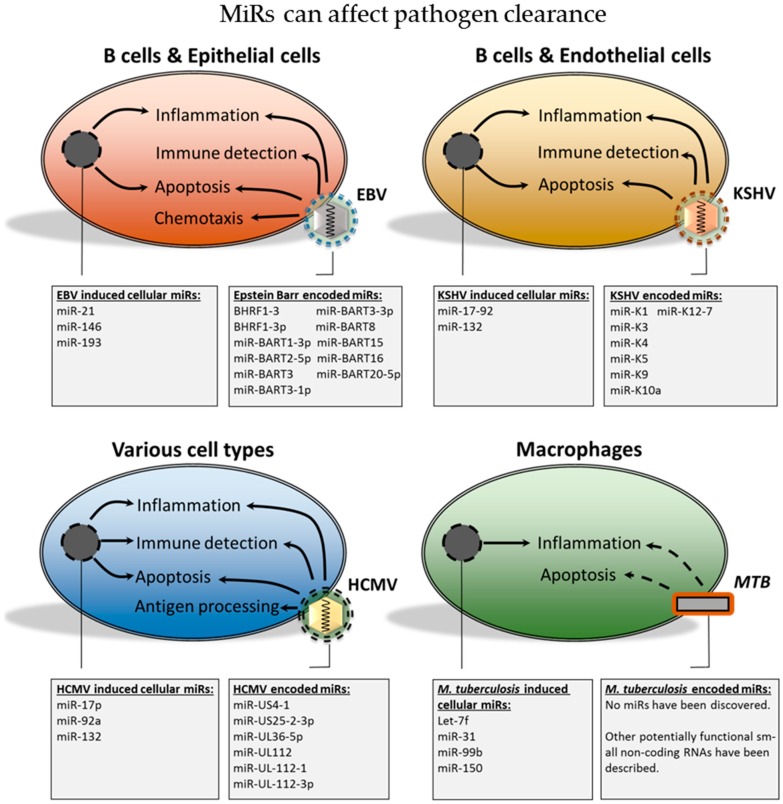
The herpesviridae have been shown to encode multiple miRs. The figure depicts the influence of these viral miRs on the immune response and viral clearance. Various analysis, including reporter assays and pull-down assays, have demonstrated that cellular mRNAs are targeted by viral miRs. This can result in decreased cellular mRNA transcription and translation of proteins, which are involved in antigen processing, inflammation, immune detection, chemotaxis and apoptosis. The figure also depicts the production of short non-coding RNAs by Mycobacterium tuberculosis (MTB), which may be relevant to its survival. Finally, host cell miRs may be induced by herpesviridae and MTB in order to shift the cellular miR expression profile and create a pathogen-permissive environment. EBV, Epstein-Barr virus; KSHV, Kaposi’s sarcoma-associated herpesvirus; HCMV, human cytomegalovirus.

Emerging evidence is suggesting that pathogens can alter cellular mRNA transcription and translation by producing miRs themselves, which has been observed in the case of some herpesviridae. Alternatively, pathogens can also induce the production of cellular miRs that downregulate immune activation, thus creating a pathogen-permissive environment. Therefore, both viral and cellular miRs may contribute to decreased pathogen clearance, which is summarized in Figure 1.

One well-known example of a pathogen that uses host cell miRs is the hepatitis C virus (HCV). Here, the cellular miR-122 can bind to the 5′-UTR of the HCV ssRNA, which promotes translation of the viral ssRNA [11,25]. In this paper we will review our current knowledge on the use and abuse of miRs by several pathogens, including the human immunodeficiency virus type 1 (HIV-1), various β and γ herpesviridae, *Mycobacterium tuberculosis* (MTB) and *Listeria monocytogenes* (*L. monocytogenes*). Furthermore, we discuss the effects on apoptosis sensitivity, detection by NK cells and cytotoxic T lymphocytes (CTLs) and increased persistence due to latency and mitigated immune signaling.

## 2. Viral miRs

### 2.1. Are miRs Relevant during Infection with HIV-1?

HIV is a single-stranded RNA (ssRNA) virus that belongs to the family of lentiviruses, which encompasses both HIV-1 and HIV-2 species. The latter has remained mostly restricted to West Africa, while the former occurs globally [26]. Most likely, chimpanzees were the initial reservoir, which then gave rise to the HIV-1 subtypes that currently causes 80% of the infections in adults via mucosal surfaces [27]. Upon infection, HIV-1 glycoprotein gp120 binds the primary CD4 receptor, which facilitates binding to the co-receptors C–C chemokine receptor type 5 (CCR5) or C–X–C chemokine receptor type 4 (CXCR4). Depending on the co-receptor expressed, HIV-1 can infect: CD4^+^ T helper (T_H_) cells, macrophages, microglia and dendritic cells [28]. Once fused with the membrane, the reverse transcriptase generates cDNA and nuclear translocation is achieved by nuclear localization signals on the proteins integrase (IN), viral proteins r (Vpr) and matrix (MA). In the nucleus, IN and chromatin-remodeling complexes facilitate the cDNA incorporation into the chromosome. Once integrated, the transcriptional transactivator (Tat) augments transcription and the mRNA is transported to the cytoplasm by the regulator of gene expression (Rev). Here, new virions are constructed from envelope (Env) proteins, ssRNA and pol with the help of negative factor (Nef), while group-specific antigen (Gag) facilitates budding of the virions [28]. Recent studies have been exploring the effects of host miRs on HIV-1 virion production and latency. In addition, while some studies detected no miR production by small genome RNA viruses, others have provided evidence that HIV-1 does express miRs [29,30,31,32,33]. In this section we address the results from novel miR detection methods, which have been used to address the validity of previously reported findings.

Whether HIV-1 also expresses miRs has been a point of discussion. Multiple studies were neither able to predict nor experimentally detect any miRs from small genome RNA viruses, while others have detected miRs [29,30,34]. The disparities between different *in silico* miR screenings are most likely the result of different miR detection algorithms and variability in parameter selection, which were used in the determination of likely candidates [32]. Yet, multiple studies reported the presence of HIV-1-derived miRs [29,30,31,32,33,35]. Some proposed that these viral miRs (vmiRs) may originate from a short transactivation response element (TAR) [36,37,38,39,40,41]. The studies, performed by Ouellet *et al.* [40], also reported the asymmetrical TAR processing by Dicer and TAR association with Ago [40,41]. Moreover, they suggested that TAR miRs influenced the balance between survival and apoptosis by regulating the translation of the pro-apoptotic Caspase 8 (CASP8) and the anti-apoptotic Aiolos. Yet, whether HIV-1 encodes additional vmiRs remains controversial, because of discrepancies in the deep sequencing data and the conflicting results reported by Pfeffer *et al.* and Lin *et al*. [30,32,34]. Both studies were unable to predict or experimentally detect any previously described functional miRs or siRNAs [36,42]

Furthermore, while recent SOLiD deep sequencing analysis confirmed the expression of miRs in the TAR, other studies disputed these findings and claimed that TAR-miRs do not associate with Ago [39,40,41]. The latter study used deep sequencing and PAR-CLIP, but found no HIV-1 miRs or siRNA, nor did they detect any vmiRs associated with Ago [43,44]. As is previously mentioned, effective mRNA degradation and translation inhibition requires miR association with Ago [5]. Therefore, it seems unlikely that the described sncRNAs from HIV-1, which are unable to associate with Ago, will elicit a significant effect on mRNA translation, or that they can be beneficial during viral latency or the lytic phase. However, some of the vmiRs have also been relieved of their potential relevance due to their low expression levels. Zhang *et al.* [33] suggested that vmiRs can target the core promoter of HIV-1 in the chromosome, which would require relatively few miR copies. In addition, they reported that miR-H3 can increase transcription of HIV-1 by increasing the association of RNA polymerase II and TATA box binding protein (TBP) to the TATA box in the 5′-LTR. Not only did miR-H3 overexpression result in a substantial increase in virion production, but siRNA binding to the TATA box was able to abolish the latency phase in resting CD4^+^ T cells [33].

While there are plenty of discrepancies in the various reported findings, a thorough analysis of the vmiR expression in cells from patients and the RISC association with host cellular miR indicated that the viral sncRNAs are expressed at low levels only and barely associate with Ago [35,38,43,44]. One explanation as to why Ouellet *et al*. [35] found miRs associated with Ago may be because they overexpressed both TAR-miRs and Ago 1,2,3 or 4 in their experiments, whereas Whisnant *et al*. [43] and Vongrad *et al*. [44] used either *in vitro* HIV-1-infected cells or patient material. We propose that the results of the latter studies add more weight as their experimental setup more closely resembles physiological conditions.

### 2.2. The Functional Role of Epstein-Barr Virus-Encoded miRs

The Epstein-Barr virus (EBV) is a double-stranded DNA γ-herpesvirus that infects B cells via binding of the envelope protein gp350 to the CD21 receptor. During primary infection, or infectious mononucleosis, the B cell population expands and causes infection of epithelial cells. EBV infection is counteracted with a CD8^+^ T cell response and CD8^+^ T memory cells provide surveillance during the latency phase [45]. During this phase, other pathologies may arise, which have been correlated with a latency program. Program I is found in patients suffering from T cell and NK cell lymphomas and correlates with the expression of Epstein-Barr virus nuclear antigen 1 (EBNA1), latent membrane protein 1 (LMP1) and variable levels of LMP2. Program II is found in Hodgkin’s lymphoma patients and correlates with EBNA1, LMP1 and LMP2 expression, while program III is found in patients that suffer from lymphoproliferative disorders and correlates to EBNA2 and LMP1 expression [46,47]. The EBNAs are involved in enhancer and promoter activation, which makes some EBNAs, like EBNA2, essential for viral transformation of B cells [46]. Besides this, LMP1 is an important protein with pleiotropic effects, which includes upregulation of adhesion molecules and the anti-apoptotic proteins A20 and Bcl-2. Due to its functional resemblance to CD40, LMP1 provides the B cell with growth and differentiation signals via MAPK and NF-κB [47]. Other latent EBV transcripts include Epstein-Barr virus-encoded small RNAs (EBER), which have been shown to bind protein kinase R (PKR) and may have a role in reducing antiviral effects of interferons [47]. Previous findings have suggested that Bam HI-A region rightward transcript (BART) miRs are evolutionarily conserved in EBV, and that these miRs may play an important role in epithelial latency [46]. This chapter reviews the reported targets of EBV-encoded miRs and discusses them according to function. The described functions are relevant for detection by the immune system, the production of cytokines, latency and apoptosis. Therefore, this section presents evidence for the involvement of EBV miRs in immune evasion and survival.

**Figure 2:**
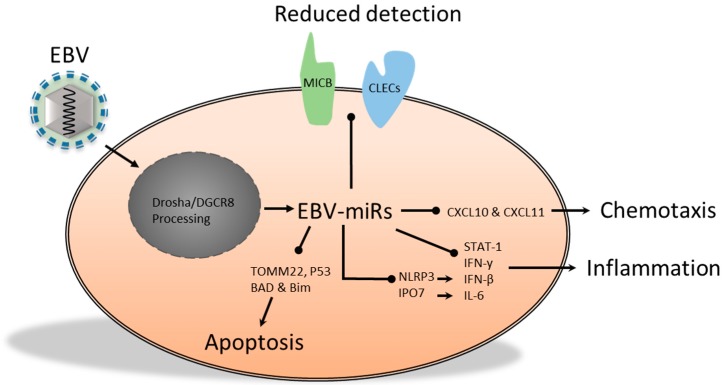
EBV miRs can alter the expression of multiple proteins, cytokines and chemokines in various cell types. These alterations may benefit pathogen persistence by affecting key pro-inflammatory processes, such as: detection by natural killer (NK) cells and cytotoxic T lymphocytes (CTLs), cytokine signaling, chemotaxis and apoptosis. MICB, CLECs, CXCL10, IPO7, Bim and TOMM22 have been verified as vmiR targets by reporter assays and PAR-CLIP analysis, while CXCL11, P53, BAD and NLRP3 have only been verified by reporter assays. MICB, MHC class I-related chain B; CLECs, C-type lectin domain family members; DGCR8, DiGeorge syndrome critical region gene 8; TOMM22, translocase of outer mitochondrial membrane 22 homolog; STAT, signal transducers and activators of transcription; BAD, Bcl-2-associated death promoter; Bim, Bcl-like protein 11; IPO7, importin 7; CXCL, chemokine (C–X–C motif) ligand; NLRP3, NLR family, pyrin domain containing 3.

EBV encodes at least 25 miRs, some of which were found to target host cell mRNAs [48]. Previously, a computational method, validated in *Caenorhabditis elegans* and *Drosophila melanogaster*, was used to predict EBV miR targets during latent infection [49]. The predicted targets showed the potential to regulate cell proliferation, apoptosis, expression of B cell-specific chemokines and cytokines, transcriptional regulators and components of signal transduction pathways [49]. Since then, more EBV miRs have been identified and analyzed, which has led to the proposition that EBV miRs may be involved in: evading immune detection, modulating immune signaling, latency and decreased sensitivity to apoptosis (Figure 2). Downregulation of LMP2A protein expression may be a way for EBV to escape immune detection, while exceeding levels of LMP1 are linked to increased sensitivity for apoptotic signals [50,51]. Targeting of LMP1 and LMP2A by the BART cluster 1 miRs and miR-BART22 has been demonstrated in nasopharyngeal carcinoma (NPC) cells, and both membrane proteins have been linked to immune activation [51,52]. In addition, PAR-CLIP analysis revealed that miR-BARTs can target the 3′-UTR of major histocompatibility complex (MHC) class I-related chain B (MICB) mRNA, which is an important ligand for NKG2D and NK cell-mediated killing [31,53,54]. However, there are some disparities in the reported types of miR-BART that are able to target the MICB 3′-UTR, which may be explained by the use of different experimental approaches. Nachmani *et al*. [53] used a computational approach and overexpression of miR-BART2-5p in order to determine its functionality, whereas Skalsky *et al*. [54] used PAR-CLIP in lymphoblastoid cell lines to identify miR-BART3 and miR-BART1-3p seed match sites in the MICB mRNA. Also, Skalsky *et al*. [54] reported the presence of miR-BART2-5p, which may suggest that this miR is functionally relevant in other cell types or in *in vivo* settings. Furthermore, Skalsky *et al*. [54] reported on miR-BARTs that could target various C-type lectin domain family members (CLEC) and other proteins including: SP100, zinc finger protein 451 (ZNF451) and phosphodiesterase 7A (PDE7A). These targets, which were validated using 3′-UTR reporter assays, may be implicated in interferon-induced antiviral defense (SP100 and ZNF451) or T cell activation (PDE7A) [54,55].

Various other miR-BARTs may affect the expression of cytokines. For example, one study found that miR-BART15 was present in exosomes and, like the cellular miR-223, was able to downregulate translation of the NLR family, pyrin domain containing 3 (NLRP3) inflammasome [56]. Uptake of these exosomes by non-infected cells reduced inflammasome activity and decreased IFN-β production, which could potentially result in mitigated viral clearance. miR-BART3, another member of the BART cluster, has been linked to decreased cytokine production as well. This BART-miR was found to associate with Ago2 in multiple lymphoblastoid cell lines and was able to target importin 7 (IPO7), which is an import receptor of c-Jun and is associated with innate immunity [13,48]. Knockdown of IPO7 in a mouse macrophage cell line resulted in decreased production of the pro-inflammatory cytokines IL-6 after lipopolysaccharide (LPS) stimulation [48,57]. Hence, miR-BART3 may influence the production of IL-6 in B cells and possibly in epithelial cells. Collectively, BamHI fragment H rightward open reading frame-1-3 (BHRF1-3), BART3 and BART15 may attenuate the immune response in EBV-infected individuals, which could aid in EBV persistence or have a potential role in induction or progression of cancer [56,58]. The effect of miR regulation in cancer is illustrated by the finding that miR-BART regulated cytokine signaling in EBV-infected nasal NK cell carcinomas. Moreover, miR-BART20-5p and miR-BART8, which were reported to downregulate IFN-γ and STAT-1 respectively, may be relevant in reducing IFN-γ-induced apoptosis during EBV infection [59].

Potential immunomodulatory functions of EBV miRs as a result of altered chemokine expression have been reported as well. The T cell-attracting chemokine chemokine (C-X-C motif) ligand 11 (CXCL-11) was found to be a target of miR-BHRF1-3 in the type III latency cell line Burkitt lymphoma-5R (BL-5R) and diffuse large B cell lymphoma (DLBCL) [48,58,60]. Moreover, Pegtel *et al.* [61] revealed that EBV-transformed lymphoblastoid B cells were able to produce exosomes that, amongst other vmiRs, contained miR-BHRF1-3 [61]. Through paracrine action, miR-BHRF1-3 reduced expression of a CXCL-11 3′-UTR reporter construct in a dose-dependent manner in HeLa cells and monocyte-derived dendritic cells (MoDCs). While this may be important to prevent recruitment of antiviral-specific T cells, miR-BHRF1-3 was not detected in the primary effusion of Burkitt lymphoma cell lines or the lymphoblastoid cell line BC-1, and further investigation of this miR *in vivo* is needed in order to elucidate its role during infection [54,58]. Furthermore, using PAR-CLIP analysis of EBV-infected lymphoblastoid cell lines, a miR-BART1-3p binding site was identified in the 3′-UTR of CXCL10 mRNA, which like CXCL-11 is an agonist for the CXCR3 receptor [54]. Both chemokines facilitate the recruitment of multiple immune cells such as type-1 helper (T_H_1) cells, cytotoxic CD8^+^ T lymphocytes (CTLs), NK cells and NKT cells [62,63]. Therefore, the expression of miR-BHRF1-3 or miR-BART1-3p may be beneficial for EBV specific to *in vivo* settings as their production may lead to decreased recruitment of CTLs and NK cells.

Latency is another way for EBV to ensure persistence and evade elimination by the immune system. Evidence that miRs promote latency was demonstrated by the finding that miR-BART2 targeted the 3′-UTR of the viral DNA polymerase BALF-5 mRNA, which is required for replication of viral genomic DNA in the late phase of lytic infection [49,64]. Since the expression level of miR-BART2 is high in latency, and BALF-5 is not expressed during latent infection, it has been suggested that miR-BART2 functions as a molecular switch to facilitate the establishment and maintenance of viral latency. Similarly, both immediate-early genes BZLF1 and BRLF1, which are essential for the induction of the lytic cycle and the reactivation of the lytic cycle from the latency phase, have been linked to miR-BART regulation in a 12-*O*-tetradecanoylphorbol-13-acetate (TPA)-activated and EBV infected human gastric adenocarcinoma (AGS) cell line [65]. In contrast to this, however, another report did not detect any miR regulation of the BZLF cluster or any miR regulation regarding latency [66]. A variety of factors may be contributing to the reported discrepancies, which include the use of different cell lines, the amount of miRs analyzed in one experiment and the activation state of the cells [65,66]. Taken together, while miR contributions may be moderate *in vitro*, EBV miRs—depending on the stage of infection—may have a role in latency by regulating BALF5.

Viral persistence can be increased by promoting host cell survival through interference with apoptosis [49]. One study suggested that BHRF1 miRs have a role in protecting primary B cells from apoptosis, though the authors did not report a cellular mRNA target [66]. More recently, Li *et al.* [67] reported that miR-BHRF1-1 increased in the NPC cell line SUNE1 after TPA stimulation and that this miR was able to target the 3′-UTR of the tumor suppressor gene p53 mRNA [67]. BHRF miRs were found to be upregulated and involved in the cell cycle during early infection, but it remains to be demonstrated whether miR regulation of p53 is involved in preventing apoptosis [66,67]. Another example of an EBV-encoded miR is miR-BART16, which was found to target the translocase of outer Mitochondrial Membrane 22 homolog (TOMM22) [13,48,68]. TOMM22 is required for association of Bcl-2-associated X protein (BAX) with mitochondria thereby promoting apoptosis [48,69]. Similarly, the Bcl-2 BH3-only family members Bcl-2-associated death promoter (BAD) and Bcl-like protein 11 (Bim) can be targeted by BART miRs. BART20-5p was shown to downregulate BAD, thereby increasing the chemotherapeutical resistance of AGS cells [70]. In addition, Bim was found to be decreased by the miR-BART cluster I, which contains eight different BART miRs [71]. Nevertheless, no single BART miR could account for the downregulation of Bim in a 3′-UTR reporter assay, suggesting that either indirect causes or miR binding to the mRNA distant from the 3′-UTR may be responsible [71]. Another report proposed that EBV miR-BART5 affected apoptosis in several EBV-infected carcinoma cell lines by targeting the 3′-UTR of p53 up-regulated modulator of apoptosis (PUMA) mRNA [72]. These cell lines were significantly more resistant to apoptosis, but knockdown of miR-BART5-augmented apoptosis induced by functional topoisomerase II inhibitors [72]. In contrast to these findings, however, others reported no or minimal inhibition of PUMA protein levels, although this was observed in an EBV non-infected gastric cell line [68,71]. In summary, vmiR regulation of BAD and PUMA remains to be elucidated; however, this regulation has been demonstrated for Bim and TOMM22, which can contribute to decreased apoptosis sensitivity.

Recently, Kang *et al.* [68] performed an extensive analysis of miRs that exerted anti-apoptotic effects in AGS gastric cancer cells using sub-G1 analysis, which is a method used to detect cells that have lost some of their DNA in the late stage of the apoptosis process following endonucleases activity. This analysis confirmed downregulation of TOMM22 by miR-BART16 and, moreover, correlated the downregulation of integrator complex subunit 6 (INTS6) expression to miR-BART3 [69,73]. Dissimilar to a previous report on EBV-associated nasal NK cell lymphoma, Kang *et al.* [68] were unable to detect miR-BART20 in AGS cells [59]. It remains unclear what could account for these discrepancies, but one possibility might be the use of different cell lines and miR detection protocols. Kang *et al.* [68] also proposed various new pro-apoptotic targets for BART2-5, BART9-3 and BART19-3p in the EBV latency II nasopharyngeal carcinoma cell line C666 cells using PAR-CLIP and antibodies against all four human Ago proteins. This, together with a 3′-UTR reporter assay, confirmed the following targets that have previously been associated with apoptosis: FEMinization-1 homolog b (FEM1B) and castor zinc finger 1 (CASZ1a) may be targets of miR-BART3, octamer binding protein 1 (OCT1) may be a target of miR-BART6, AT rich interactive domain 2 (ARID2) may be a target of miR-BART8, cAMP response element-binding protein binding protein (CREBBP) and SH2B3 may be targets of miR-BART16 and protein phosphatase 3 regulatory subunit B alpha (PPP3R1), P21 activated kinases (PAK2) and tumor protein p53 inducible nuclear protein 1 (TP53INP1) may be targets of miR-BART22 [68]. The targets from this reporter assay and PAR-CLIP may therefore contribute to decreased apoptosis sensitivity.

In summary, the studies discussed above indicate that miRs expressed by EBV affect the translation of cellular proteins. Some of these targets have been confirmed by reporter assays and PAR-CLIP, which together are a good indication that these EBV miRs may also be relevant in an *in vivo* setting. Overall, EBV-derived miRs possess the potential to promote latency. In addition, it seems that EBV miRs are involved in mitigating the immune response and abating pro-apoptotic signals. This may provide the virus with additional mechanisms to increase its potential for persistence and survival.

### 2.3. miRs Produced by the Kaposi’s Sarcoma-Associated Herpesvirus

**Figure 3:**
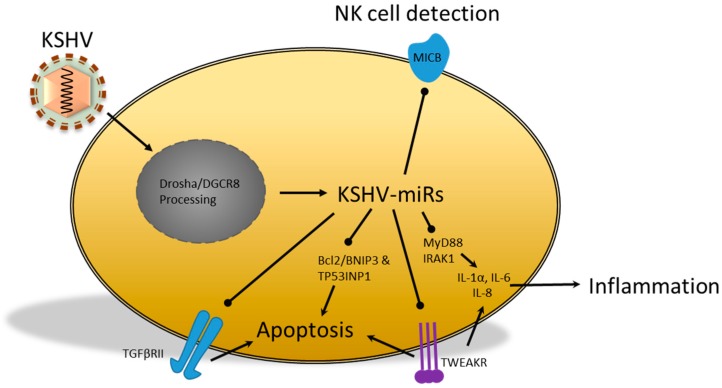
KSHV-encoded miRs that are able to regulate cellular targets, some of which have been shown to regulate the transcription of cytokines or their receptors, including the TGFβRII. Furthermore, KSHV miR inhibition of pro-apoptotic factors, together with reduced NK cell detection, may contribute to cell survival and increased viral persistence, cytokine production, cytokine signaling, NK cell detection and apoptosis in various cell types. The vmiR targeting of MyD88, IRAK1, TGFβRII and MICB has been demonstrated in reporter assays, while TWEAKR, Bcl-2/BNIP3 and TP53INP1 have been verified by PAR-CLIP analysis. TWEAKR, tumor-necrosis factor-related receptor; IRAK1, Interleukin-1 receptor-associated kinase 1; BNIP3, BCL2/Adenovirus E1B 19kDa Interacting Protein 3; TP53INP1, Tumour protein p53 Inducible Nuclear Protein 1; MyD88, myeloid differentiation primary response protein.

Like EBV, Kaposi’s sarcoma-associated herpesvirus (KSHV) is a γ-herpesvirus and is tropic for B cells and endothelial cells, since KSHV DNA can be detected in spindle cells *in vivo* [74]. KSHV infection is required for the development of Kaposi’s sarcoma (KS) and this type of cancer can occur in the absence of HIV-1 infection or it can coincide with HIV-1 and AIDS. Typically, KS is confined to the skin and occurs most frequently on the legs, while AIDS-associated KS is more malignant and can be more widespread [74]. The virus encodes at least 12 miRs, most of which are located between v-FLice-inhibitory protein (v-FLIP) and the long inverted repeat 1 (LIR) [31,74] signaling. This chapter reviews the cellular processes, involved in fighting KSHV infection, which may be subjected to vmiR regulation. We will discuss the role of KSHV vmiRs as potential immunomodulators, which may be involved in immune detection and cytokine production. In addition, we discuss the findings that suggest a potential for a vmiR-directed decrease in apoptosis sensitivity. The collective effects of KSHV vmiRs is summarized in Figure 3. More than seven KSHV miRs have been found to downregulate cellular targets [53]. As with EBV, KSHV miRs regulated various cellular processes, which included immune detection, immune activation and apoptosis (Figure 3). Detection by the immune system could be mitigated by miR-K12-7 due to downregulation of MICB [53]. Not only was this demonstrated in a reporter assay, but also in a physiologically more relevant setting, which involved KSHV-infected body cavity-based lymphoma (BCBL1) cells. In addition, NK cell-mediated killing was significantly reduced in infected BCBL1 cells when compared to cells transduced with anti-miR-K12-7, suggesting that MICB downregulation might contribute to the immune evasion strategy of KSHV [53]. Furthermore, signal transducers of the immune response were downregulated by KSHV. More specifically, the mRNA of the TLR adaptor protein myeloid differentiation primary response protein (MyD88) has a 3′-UTR target site for miR-K5, while the kinase IRAK1 mRNA was targeted by miR-K9 [31,75]. This inhibited NF-κB activation in IL-1α-stimulated endothelial cells and TLR7/8-stimulated B cells. Co-transfection of both miR-K5 and miR-K9 in epithelial cells resulted in reduced IL-6 and IL-8 mRNA and protein levels, which is expected to reduce inflammation and neutrophil recruitment [75]. Other studies have found that the KSHV-encoded E3 ligase replication and transcription activator (RTA) was able to degrade MyD88 via ubiquitination and proteasomal degradation during early infection [76]. The authors suggested that RTA may be of importance during the early stages of infection and that miR-K5 might be important during later stages or latency [76]. This notion is supported by the finding that miR-K5 was upregulated during later stages of infection and that MyD88 was able to inhibit latency-associated nuclear antigen-1 (LANA-1) [76]. LANA-1 facilitates the replication of the viral episome and is important for persistent infection [74]. Therefore, these studies indicate that KSHV miRs may decrease NK cell recognition and attenuate TLR signaling, which could decrease cytokine production and promote latency.

Previously, multiple vmiRs have been linked to increased resistance to apoptosis. In a recent study, Gottwein *et al*. [13] analyzed the association of KSHV RISC with mRNAs in primary effusion lymphoma (PEL) cell lines using PAR-CLIP. They identified over 2000 potential cellular mRNAs, which included the pro-apoptotic proteins BCL2/Adenovirus E1B 19 kDa interacting protein 3 (BNIP3) and the previously mentioned TP53INP1 [13,68]. Amongst the potential targets was tumor-necrosis factor-related receptor (TWEAKR), which could be downregulated by miR-K10a [77]. This downregulation was observed in lymph node biopsies from KS patients and miR-K10a overexpression in HUVECs. Functionally, miR-K10a overexpression in SLK cells, a Kaposi’s sarcoma-derived cell line, revealed decreased CASP-mediated apoptosis [13,31,77,78,79]. Besides this, miR-K10a has been linked to direct and indirect alterations in cytokine expression. The inhibition of TWEAKR by miR-K10a resulted in decreased signaling and reduction of IL-8 and CCL2 in response to TWEAK. Abend *et al*. [77] suggested that KSHV may balance expression of these cytokines to levels that are not detrimental to the virus. In a more recent study, the KSHV infection of telomerase-immortalized microvascular endothelial (TIME) cells was correlated to an 86% decrease in TGFβRII protein levels [80]. This reduction was attributed to miR-K10a, as several cell lines expressed decreased TGFβRII protein levels. Due to the TGFβRII downregulation, miR-K10a was able to protect cells from increased apoptosis by reducing cellular TGF-β sensitivity. In summary, there is some evidence that KSHV miRs are able to reduce immune detection and TLR signal transduction. Others have used PAR-CLIP to identify vmiR targets, which indicates that sensitivity to apoptosis may also be affected. Future studies will have to elucidate how important the contributions of these vmiR are for KSHV survival.

### 2.4. Human Cytomegalovirus; miRs in Persistence and Immunomodulation

**Figure 4:**
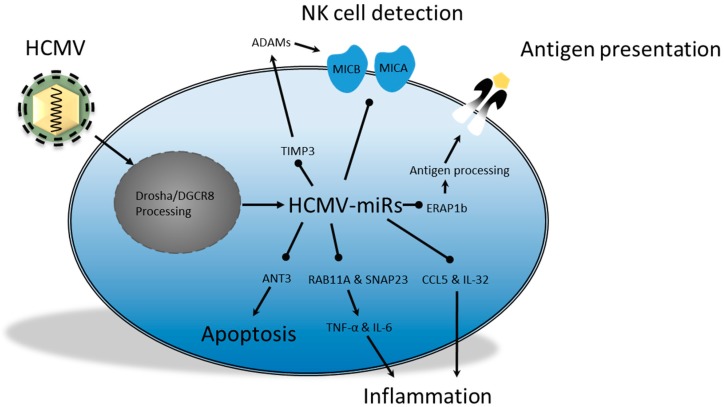
HCMV-encoded miRs downregulate targets that are involved in modulating the production and secretion of pro-inflammatory cytokines. In addition, these vmiRs can promote MICA/B shedding by reducing the negative regulator TIMP3, which results in decreased NK cell detection. Detection of HCMV-infected cells by CTLs can also be decreased due to mitigated antigen processing as a result of reduced ERAP1. TIMP3, MICA/B, ANT3, IL-32 and CCL5 targets were determined using reporter assays, while RAB11A and SNAP23 targets were determined using RIP-CHIP and the ERAP1b target was determined using RISC immunoprecipitation. ADAMs, a disintegrin and metalloprotease; TIMP3, tissue inhibitors of metalloproteinase 3; ANT3, adenine nucleotide translocator 3; RAB11A, RAS-related protein 11A; SNAP23, synaptosomal-associated protein 23; ERAP1b, endoplasmic reticulum aminopeptidase 1b.

The human cytomegalovirus (HCMV) is a β-herpesvirus that spreads through contact with bodily fluids and then causes persistent infection. The virus has broad tropism and can cause dangerous pathologies in immunocompromised patients, while infection in pregnant women increases the risk of congenital defects. The process of HCMV adhesion is still being elucidated, though it is likely to involve the binding of cellular integrins by the glycoproteins gB, gH/gL and various uncharacterized proteins, including: UL128, UL130 and UL131 [81]. After fusion, the extensive 230 kb dsDNA is transcribed, which includes the transcription of multiple acquired homologs of host genes. These homologs include, amongst other factors, various unique short (US) glycoproteins, which can induce degradation of MHCI and of some MHCII molecules, retain MHCI in the endoplasmic reticulum (ER) and interfere with MHCI peptide loading [82]. The discovery that some vmiR regulation may be advantageous during immune evasion has also fueled the suspicion that HCMV may be employing miRs for similar purposes. This chapter reviews the cellular processes, involved in fighting HCMV infection, which may be subjected to vmiR regulation. It describes the alterations in protein expression and the effects this has on immune detection, PAMP sensing, cytokine signaling and apoptosis. The collective effect of HCMV vmiRs is summarized in Figure 4.

Previous studies have demonstrated that HCMV miR could inhibit the expression of cellular targets involved in CTL and NK cell activation. One way to decrease CTL activation is by downregulation of the antigen processing, thereby preventing antigen loading on MHCI molecules. Kim *et al*. [83] found that one of the protein-processing enzymes, the amino peptidase endoplasmic reticulum aminopeptidase 1b (ERAP1b), was downregulated by miR-US4-1. Furthermore, miR-US4-1 was found in association with Ago and its target mRNA sequence. This, together with the fact that transfection of synthetic miR-US4-1 in fibroblasts from HCMV-seropositive donors resulted in decreased cell lysis by five different CTL clones, reinforced the notion that HCMV can specifically act to manipulate immune activation. The suggestion that HCMV can manipulate immune activation is further supported by observations that HCMV affects NK cell activation. Decreased activation of NK cells via NKG2D has previously been correlated with the downregulation of stress-induced ligands like MICA and MICB, which can be cleaved by metalloproteinases to decrease NKG2D-mediated NK cell recognition of target cells [84]. A recent report by Esteso *et al*. [84] indicated that the viral miRUS25-2-3p may downregulate expression of tissue inhibitors of metalloproteinase 3 (TIMP3), which is associated with the inhibition of a disintegrin and metalloprotease (ADAM) activity. Similarly, the vmiR miR-UL112 was found to target the 3′-UTR of MICB mRNA, which resulted in translational repression. This specifically inhibited the NKG2D NK cell-mediated killing of HCMV-infected human colorectal carcinoma (RKO) cells and was likely independent of the known posttranslational MICB viral regulator UL16 [53,85]. A more recent paper by Nachmani *et al.* [86] demonstrated that these viral miRs from HCMV, EBV and KSHV did not randomly target the 3′-UTR of the MICB mRNA [86]. In fact, binding sites of vmiRs overlapped with endogenous cellular miRs. Therefore, they suggested it may be difficult for the cell to escape vmiR regulation, since mutations in this region will make the cell more susceptible to NK cell-mediated killing. Moreover, miR-UL112 was able to act synergistically with the cellular miR-376a; both were able to decrease MICB expression and their ablation resulted in increased NK cell-mediated killing [86]. This suggests that it may be favorable for vmiRs to target endogenous miR binding sites, as they are likely to be conserved and have the potential to enhance vmiR efficacy. Moreover, these results support the idea that vmiRs, in addition to other HCMV immune evasion mechanisms, may contribute to decreased detection of infected host cells.

The potential of HMCV miRs to interfere with the sensing of PAMPs is currently being elucidated. UL-112-3p was able to target two 3′-UTR sites of TLR2 mRNA and possibly other members of the NF-κB pathway like IRAK1 [87]. The authors further substantiated the relevance of these findings by considering previous reports, which found that gB and gH are ligands for TLR2 and that TLR2 KO mice, infected with murine CMV, expressed lower IL-18 and IFN-α/β levels combined with a decrease in NK cells numbers [87,88,89]. Yet, it appears that TLR2 is not downregulated until late stages of the lytic cycle, which raises the question of the importance of UL-112-3p in downregulating the innate immune response [87]. Moreover, while miRs may act in a paracrine fashion via exosomes, these would have to reach specific cells with sufficient quantities of miR UL-112-3p in order to function as paracrine modulator of TLR2 expression, which has yet to be demonstrated. Nevertheless, Nachmani *et al.* [86] previously demonstrated that the miR expression pattern of the infected cell type may result in a synergistic effect [86]. Therefore, it could be hypothesized that HCMV might be able to affect TLR expression in close vicinity of the infected cell, thus attenuating the innate immune response from the infected tissue. Other indications that HCMV miRs are involved in the downregulation of the immune response comes from the recent finding that CCL5 production by fibroblasts is impaired during infection with the virulent clinical HCMV strain Toledo [90]. This strain produces miR-UL148D, which was linked to decreased CCL5 mRNA and protein levels. However, whether this downregulation occurs *in vivo*, and whether it will influence the recruitment of leukocytes remains to be investigated. One study did find a disparity in IL-32 expression in actively infected patients, and patients that had been infected by the virus in the past. This report found that miR-UL-112-1 was able to decrease IL-32 mRNA and protein levels after the immediate early stage of infection [91]. Virally induced alteration of IL-32 expression may be beneficial for the virus as it could mitigate inflammation by decreasing the production of TNF-α. In addition to decreased cytokine production, reduced cytokine secretion may influence the antiviral response as well. Evidence that the HCMV encodes miRs that interfere with the endocytic and/or exocytic pathway has recently been demonstrated in a RIP-CHIP analysis [92]. The mRNAs of vesicle-associated membrane protein 3 (VAMP3) and RAS-related protein 5C (RAB5C) were enriched in HEK293T cells after a miR-UL112-1 RISC immunoprecipitation and DNA microarray. Furthermore, reporter assays revealed that miR-UL112-1 and miR-US5-1 could target the 3′-UTR of the mRNA from exocytotic proteins RAS-related protein 11A (RAB11A) and synaptosomal-associated protein 23 (SNAP23), while miR-US5-2 reduced the expression of SNAP23. Analysis of the supernatant from THP-1 cells, transfected with all three miRs, revealed an 8-fold decrease in TNF-α levels and an approximate 2-fold reduction in IL-6 levels. Thus, these vmiRs target multiple mRNAs from proteins involved in endo- and exocytosis, thereby supporting a phenotype with decreased immune activating capabilities. Overall, these reports suggest that the HCMV virus can produce vmiRs that have the potential to downregulate innate immune activation and the recruitment of immune cells. Future studies will have to determine to what degree these vmiRs may contribute to viral persistence *in vivo*.

Like the γ-herpesviruses, HCMV may also employ vmiRs to increase cellular resistance against apoptosis. Recently, Babu *et al.* [93] used multiple miR target prediction algorithms to predict the binding of HCMV-miRs to the 3′-UTR of cellular mRNAs [93]. They predicted that miR-UL70-3p may target modulator of apoptosis 1 (MOAP1) and miR-UL70-3p may target putative HLA-DR-associated protein 1 (PHAP1), both of which are associated with apoptosis. However, the functional relevance of these vmiRs has yet to be demonstrated. Another HCMV vmiR that was associated with decreased sensitivity to apoptosis was miR-UL36-5p. This vmiR was able to target adenine nucleotide translocator 3 (ANT3) in human embryonic lung fibroblasts (HELF) [94]. More importantly, directly after HCMV infection of HELF cells, the ANT3 levels were significantly downregulated. Over time, the level of ANT3 expression still increased, though considerably less than cells transfected with a non-specific miR. Furthermore, overexpression of miR-UL36-5p decreased apoptosis in different cell lines, while the antagomir abolished this effect. This study indicates that miR-UL36-5p may be an important inhibitor of apoptosis during early infection, though the importance of this function during lytic replication and latency remains to be demonstrated [94].

In summary, HCMV is one of the herpesviruses that produces vmiRs. Like EBV and KSHV, HCMV-derived miRs have been linked to decreased immune detection, interference with cytokine signaling and have the potential to affect the induction of apoptosis. Admittedly, a lot of these effects are mild, though HCMV miRs may alter cellular functions, which ultimately benefit the persistence of the virus. Based on such observations, the role of vmiRs in establishing a cellular-permissive phenotype should not be underestimated. Further analysis of their significance *in vivo*, however, is required in order to determine their importance.

## 3. Host Cell miRs: A Means for Viral Immune Modulation?

Thus far we have reviewed our current knowledge on miRs expressed by different viruses and the consequences for viral persistence. However, viruses may also induce expression of endogenous miRs in target cells that consequently affect the functionality of this cell or allow for viral persistence. The hypothesis that pathogens may benefit from the induction of specific cellular miRs is derived from observations that specific host cell proteins and antigens are capable of inducing miRs, which then affect innate and acquired immune activation. Moreover, the finding that cellular miRs may be of benefit to viruses is demonstrated in the case of HCV. This positive ssRNA hepacivirus contains a 5′-nontranslated region (NTR) that contains a type III internal ribosome entry site. Ribosome association occurs without a 5′-RNA cap, but instead the 5′-NTR associates with cellular miR-122, which enhances stabilization of the viral RNA [11]. The significance of stabilization was enforced by the finding that knockdown of miR-122 resulted in a drastic decrease in viral titers [95,96]. miRs of similar importance to other viruses, such as HIV-1 and herpesviridae, have yet to be identified, though alteration of cellular miR transcription has been linked to decreased induction of apoptosis, reduced inflammation and increased immune evasion. In this section we will review our insights of cellular miRs that can be induced by HIV-1 and herpesviridae and affect immune responses, thereby increasing viral persistence.

### 3.1. Cellular miR Expression; a Way for HIV-1 to Sustain Latency?

An indication that cellular miRs may have a role in regulating virion production comes from knockdown experiments in which expression of miR processing and silencing enzymes were inhibited. Triboulet *et al*. [97] reported that knockdown of Dicer and Drosha in peripheral blood mononuclear cells from HIV-1-infected donors resulted in a significant increase in HIV-1 titers. Later, the same group not only reported that knockdown of Drosha and DGCR8 increased viral production, but that HIV-1 mRNA also physically associated with Ago2 [98]. In addition, Ago2 co-localized with DEAD box protein p54 in P bodies, and knockdown of DGCR8 or p54 resumed viral production in peripheral blood monocytes. Together, these studies support the notion that the mRNA silencing machinery has a relevant role in HIV-1 latency, though these claims remain controversial. This section reviews two cellular miRs that may target HIV-1 mRNA translation and that have been linked to alterations in HIV-1 virion production.

Recently, Whisnant *et al.* [43] analyzed the differential changes in cellular miR profiles of HIV-infected *versus* non-infected cells and found only minimally altered miR expression in several cell lines [43]. No significant alterations in previously described miRs, like the miR-17/92 cluster, were observed [97]. miR-29, another vmiR that was analyzed by Whisnant *et al.* [43], was able to associate with RISC in C8166 cells, but failed to downregulate Nef in a reporter assay. Yet, the possibility was raised that in the context of very high miR-29 or very low Nef expression the impact of this regulation might be significant. Others reported that miR-29 overexpression or knockdown had a significant impact on the viral mRNA levels and on virion production [99,100]. miR-29 overexpression downregulated Nef and Gag-related mRNAs, thereby decreasing viral amplification. Furthermore, miR-29a was co-purified with Gag mRNA and P body protein p54. In line with this, knockdown of p54, Ago2 or decapping mRNA 2 (DCP2) resulted in enhanced HIV-1 production and infectivity [9,100]. Recently, Adoro *et al.* [101] demonstrated that miR-29 expression significantly increased in CD4^+^ T cells upon STAT3 phosphorylation induced by either IL-21, IL-6 or IL-10. Interestingly, an inverse correlation between plasma IL-21 levels and viral titers were observed during early HIV-1 infection in bone marrow–liver–thymus (BLT) humanized mice [101]. Future studies will have to assess to what extent IL-21-induced miR-29 upregulation can contribute to limiting HIV-1 in early stages of infection. Activated CD4^+^ T cells also expressed high levels of miR-155 [102], which, when co-localized in a RISC complex, was able to reduce the number of HIV-1 transcripts [43]. This may be the consequence of translation inhibition of tripartite motif-containing protein 32 (TRIM32), which interacts with the activation domain of the HIV-1 Tat protein, as miR-155 was shown to target TRIM32 mRNA in a T lymphocyte cell line that developed post-integration latency [103]. This was reinforced by the finding that knockdown of Dicer and DGCR8 increased TRIM32-mediated ubiquitinylation of IκBα, resulting in activation of NF-κB and enhanced HIV-1 transcription. Together this suggested that increased TRIM32 may induce HIV-1 virion production during latency, while miR-155 could contribute to re-silencing of the virus and maintenance of the latent reservoir.

Collectively, these studies indicate that alterations in endogenous miR expression may have a relevant role in promoting and maintaining HIV-1 latency. These findings are of interest since depletion of the viral reservoirs, in combination with highly active antiretroviral therapy (HAART), may be an approach to eradicate HIV-1 [102].

### 3.2. Herpersviridae and Dysregulation of Cellular miRs

**Figure 5:**
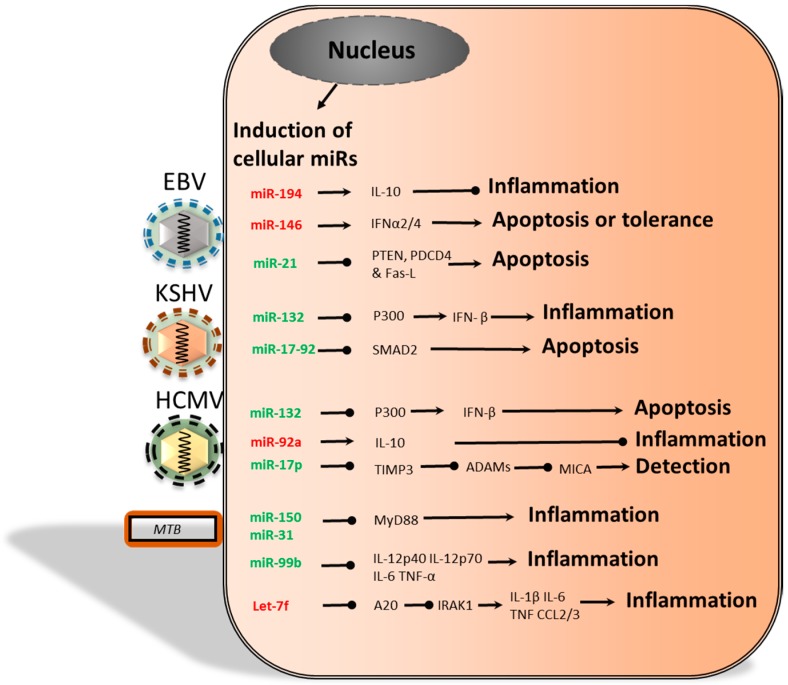
The decrease (red) or increase (green) of a particular miR, during infection, may alter the mRNA translation of specific cellular proteins. Altered protein expression can interfere with inflammation, apoptosis or NK cell detection, which may benefit the pathogen. First, EBV-induced miRs may reduce inflammation and apoptosis, but may also stimulate IFNα2/4, which could promote apoptosis or tolerance. Second, KSHV-induced cellular miRs may interfere with IFN-β and SMAD signaling, thereby affecting inflammation and apoptosis. Third, the HCMV-induced decrease of miR-92a could reduce inflammation by increasing IL-10, while miR-132 and miR-17p can affect apoptosis and detection by NK cells. Finally, the MTB *-*induced miRs have all been associated with decreased inflammation. PTEN, phosphatase and tensin homolog; SMAD2, small mothers against decapentaplegic 2; PDCD4, programmed cell death protein 4; Fas-L, Fas ligand.

Analysis of various cellular miRs have demonstrated that their expression can be altered by herpesviridae (Figure 5). The mechanistic elucidation of altered miR expression has been an ongoing line of research. Various viral proteins expressed by EBV and HCMV are likely to be involved, in order to increase the levels of anti-inflammatory cytokines, while KSHV target cell binding was sufficient to decrease cytokine transcription. This section explores the effects of herpesviridae on cellular miR expression in further detail. This section explores the alteration in gene transcription by EBV, KSHV and HCMV. More specifically, we discuss the proposed mechanisms that induce changes in host gene transcription during EBV or KSHV infection and the possible alterations in immune functions that are linked to HCMV infection.

Previously, LMP1 signaling, during EBV-type III latency programs, was found to induce NF-κB-dependent expression of miR-146a in three different lymphoblastoid cell lines [104]. It was noted that the latency cell lines expressed significantly higher miR-146a than the EBV-negative cells that overexpressed LMP1. Later, Rosato *et al*. [105] demonstrated that in the latency III U2932 B cell lymphoma cell line, the increased expression of miR-146a by LMP1 can be ablated by EBNA2. Consequently, levels of the miR-146a target IRAK1 were elevated, which was linked to increased TLR signaling and innate immune activation. The antiviral IFNα2 and IFNα4 were upregulated as well, so it seems that the downregulation of miR-146a may be more beneficial to the host rather than an immunomodulatory strategy of the virus. Nevertheless, Rosato *et al*. [105] proposed that prolonged IFN production may result in increased tolerance to IFNα, which is beneficial to EBV, since type I IFN is associated with increased apoptosis and cellular antiviral mechanisms [106]. Conversely, increased IL-10 has recently been associated with decreased apoptosis of B cell lines obtained from patients who developed posttransplant lymphoproliferative disorders (PTLD) [107]. In these patients, LMP1-induced signaling resulted in a significant reduction of miR-194, a cellular miR that has been shown to target the 3′-UTR of IL-10 mRNA. Overexpression of miR-194 resulted in decreased IL-10 levels and increased apoptosis of four spontaneously derived B lymphoblastoid cell lines. Therefore, both LMP1 and EBNA2 can, under certain circumstances, modulate pro-apoptotic signals induced by cytokines. In addition, both LMP1 and EBNA2 have also been linked to the targeting of intracellular pro-apoptotic proteins. For example, each of these proteins have been shown to induce expression of miR-21, which can target the 3′-UTR of mRNAs of various apoptosis-related proteins like phosphatase and tensin homolog (PTEN), Fas ligand (Fas-L) and programmed cell death protein 4 PDCD4 [105,108]. Together, these studies indicate that EBV alters the expression of various cellular miRs, which have a role in immune activation and regulation of pro-apoptotic signaling.

Distinct patterns in cellular miR expression have also been discovered in patient-derived Kaposi’s sarcoma (KS) cells, which are mesenchymal tumor cells of endothelial origin [109]. *In silico* predictions revealed that miR-513a-3p may target secretory phospho-protein-1 (SPP1), also called osteopontin (OPN), which may have a role in the upregulation of IFN-γ and IL-12. Let-7 family members and miR-98 can target the pro-apoptotic CASP3 and miR-17 family members can target TGF-βRII, which may both affect growth and differentiation, as well as increase apoptosis. Recently, the miR-17-92 cluster has also been correlated with the downregulation of TGF-β signaling in the endothelial cell line SLK and epithelial cell line TIVE-LTC. Both the viral latency proteins v-FLIP and v-Cyclin were able to drastically upregulate this miR cluster, resulting in decreased transcription and reduced protein levels of SMAD2. This reduction directly affected TGF-βR sensitivity, which during latent KSHV infection could protect the host cell from apoptosis [110]. Furthermore, the binding of KSHV virions has been shown to induce the transcription of miRs [111]. This induction has been linked to cyclic AMP-response-element-binding (CREB) protein-mediated activation of miR-132 transcription. The produced miR-132 was capable of downregulating the transcriptional co-activator P300, which was correlated with decreased H3K18 acetylation and reduced mRNA levels of IFN-β, ISG15, IL-1β and IL-6. Together, reducing the production of these cytokines may provide KSHV with a means to mitigate the innate immune response during the initial stages of infection.

Like KSHV, also HCMV increased expression of miR-132 in THP-1 cells, while inhibition of miR-132 resulted in decreased viral titers [111]. A mitigated immune response due to decreased levels of IFN-β may have a significant role in elevated viral titers [111]. Another way for HCMV to diminish the immune response may be by reducing hsa-miR-92a expression, which was shown to be downregulated by the HCMV IL-10 homolog LAcmvIL-10 in CD14^+^ myeloid cells. As a consequence, low hsa-miR-92a expression levels resulted in increased production of IL-10 and CCL8 [112]. Similarly, increased IL-10, CCL8 and TGF-β production have been observed in the latency-associated secretome of CD34^+^ hematopoietic progenitor cells. This secretome was able to decrease the production of IFN-γ, TNF-α and TNF-β by T_H_1 cells, while antibodies directed against IL-10 and TGF-β restored IFN-γ production [113]. Therefore, LAcmvIL-10 not only has a role in mitigating the host’s antiviral response but, by increasing CCL8, may promote the recruitment of CD4^+^ T cells. How this recruitment could benefit HCMV is unclear, though Mason *et al*. [113] speculated that this may be a side effect of the viral latency-associated secretome. As previously mentioned, viral detection can also be affected by the vmiR miRUS25-2-3p, which downregulated expression of MICA. In addition, HCMV induces expression of the cellular miR-17p during early infection of U373 cells [84]. Both these viral and cellular miRs have been correlated to 3′-UTR mRNA binding and downregulation of TIMP3, which may have an important role in the shedding of MICA from the membrane. The relevance of MICA/B shedding *in vivo* was demonstrated by the observation that HCMV-infected transplant patients had increased MICA plasma levels compared to patients who had received antiviral medication [84].

In summary, it seems that during EBV and KSHV infection, regulation by vmiRs can directly or indirectly influence pro-apoptotic signaling and cytokine levels. Similarly, HCMV may mitigate the immune response by increasing IL-10 and recognition by NK cells. Together, vimiRs and specific host miRs may function in concert to decrease the host’s antiviral response.

## 4. Bacterial and Cellular miRs

Unlike the current elucidation of vmiR production and their targets, secretion of miRs by bacteria has not been subjected to comparable levels of inquiry. Recently, one study addressed this issue and investigated the production of miRs by intracellular bacteria, which included *Chlamydia trachomatis*, *Legionella pneumophila*, *Mycobacterium marinum* (*M. marinum*) and MTB [114]. Notably, with the exception of one potential miR expressed by *M. marinum*, no miRs of bacterial origin have been detected. Nevertheless, this study did describe that MTBproduced high levels of several sncRNAs, which were detected in human THP-1 cells and recovered from the lungs of infected mice. Similarly, RNA fractions from MTBhave previously been linked to a reduced ability of monocytes to control bacterial growth as these RNAs induced intracellular TNF-α production in human monocytes, which activated CASP 8 and monocyte apoptosis [115]. Admittedly, these sncRNAs are not conventional miRs and their mode of action remains speculative. Alternatively, changes in the host’s endogenous miR expression levels have been reported. The next section discusses these reports and makes the distinction between the studies that found alterations during infection and those that attempted to determine if the alterations are pathogen specific. This is of importance since pathogen-specific miR expression profiles could be of therapeutic interest.

### Cellular miRs Induced by Bacteria

The importance of cellular miRs for the clearance of bacterial infections has previously been demonstrated. More specifically, miR-155 expression has a role in nasopharyngeal clearance of *Streptococcus pneumoniae* and the clearance of *Helicobacter pylori* in mice [116,117]. Moreover, miR-29 was downregulated upon *L. monocytogenes* and *Mycobacterium bovis* (*M. bovis*) BCG infection, which resulted in increased IFN-γ production by NK cells and T_H_1 cells [118]. In addition, murine miR-29 knockout strains revealed increased IFN-γ and lower bacterial burden, thus signifying the importance of cellular miRs and their potential as therapeutic targets. Moreover, induction of cellular miRs has been linked to alterations in immune activation, which may be advantageous to bacterial survival. Such cases have been reported and include, amongst others, bacteria like *L. monocytogenes* and MTB [119,120]. In this chapter we review the effects of listeriolysin (LLO) on host miR expression and MTB-induced alterations in immune signal transduction, which may partially be pathogen specific.

First, *L. monocytogenes* is a gram-positive motile spirochete that poses a particular threat to immunocompromised individuals. Infection with this bacterium usually occurs when people come in contact with contaminated food [121]. Typically, the bacterium infects the epithelial cells of the small intestine, followed by infection of phagocytes and Peyer’s patches [122]. Then, *L. monocytogenes* can potentially migrate, with the help of phagocytes, to secondary organs like the spleen and the liver. The phagocytosed bacteria can avoid digestion by producing LLO, an essential virulence factor, which facilitates the escape from the phagosome [123]. Some evidence suggested a role for LLO in altering host miR expression. Stimulation of caco-2 endothelial cells with LLO resulted in upregulation of miR-16, which may be involved in degrading TNF-α, IL-6 and IL-8 transcripts [124]. Furthermore, miR-146b was also upregulated by LLO, and this may in some cases be linked to decreased transcription of IL-8 and hence reduced neutrophil recruitment [124]. In addition, LLO-induced downregulation of miR-145 may increase IFN-β expression, while at the same time reducing the pro-inflammatory cytokines IL-5 and IL-13 [124,125]. Future studies will have to clarify whether the LLO-induced miRs are beneficial for the persistence of *L. monocytogenes*.

Second, MTB is an aerobe, acid-fast and facultative intracellular bacillus that is characterized by slow growth and resistance against antimicrobial substances. Initial infection with this bacterium results in recruitment of monocytes and macrophages, which encapsulate the growing bacteria, thus forming a tubercle. Multiple studies have indicated that miR regulation can have beneficial effects on MTB survival. For example, miR-124 was found to be upregulated in peripheral leukocytes isolated from MTB-infected patients [126]. In addition, miR-124 was upregulated in a *M. bovis* BCG-infected macrophage cell line (Raw264.7) and repressed the inflammatory response from alveolar macrophages. The effect of miR-124 upregulation was linked to translational regulation of TLR6, MyD88, TRAF6 and TNF-α. This suggests that miR-124 might act as a negative feedback regulator in TLR signal transduction, which may be of benefit to MTB during infection [126]. Similarly, TLR signal transduction can, during MTB infection, also be reduced by miR-146a-mediated downregulation of TRAF6 and IRAK1, which has been linked to reduced translation of TNF-α, IL-1β, IL-6 and MCP-1 in Raw264.7 cells [127,128]. Another study found upregulation of miR-132 and miR-26a in monocyte-derived macrophages (MDM) during early MTB infections. The data suggested that these miRs downregulated P300, which partially functions as histone acetyl transferase (HAT). In the nucleus, this activator can associate with STAT-1, and downregulation of P300 can therefore result in impaired activation of IFN-γ response genes. Evidence of altered IFN-γ response gene activation was demonstrated by the observation that FcγRI and HLA-DR were decreased in MTB*-*infected MDMs [129]. Thus, a part of the MTB survival strategy might be the downregulation of IFN-γ signaling, which results in less efficient phagocytosis and decreased antigen presentation. Even though the studies discussed above present evidence of miR regulation that downregulates immune activation, it has not been investigated whether the upregulation is merely part of a negative feedback mechanism employed to fine tune downstream TLR gene transcription. Therefore, whether these miRs are actively involved in the survival strategy of MTB remains to be elucidated. However, even if these miRs turn out to be part of an endogenous negative feedback mechanism, they should not be disregarded as potential therapeutic targets, since alterations of specific miR concentrations could increase host pathogen clearance [118].

More recent studies have demonstrated that MTB can also specifically alter cellular miR expression in a manner that inhibits immune activity in infected cells, but not in cells that were challenged with control antigens or other bacteria (Figure 5). One study found the upregulation of sonic hedgehog (SHH) in pulmonary TB patients [130]. Subsequent miR expression profiling of PBMCs revealed that miR-31 and miR-150 levels were significantly increased in TB patients compared to healthy individuals. In line with this, both miRs were also upregulated by *M. bovis BCG* infection of mouse PBMCs, which was dependent on SHH and TNF-α. miR-31 and miR-150 inhibited MyD88 translation, which led to abated downstream TLR2-induced transcription of pro-inflammatory genes. Together, the effect of this downregulation may contribute to an MTB-permissive environment. Another example is miR-99b, which is upregulated to high levels in MTB-infected murine DCs compared to LPS-stimulated DCs [131]. miR-99b knockdown experiments revealed reduced bacterial growth and significant upregulation of IL-12p40, IL-12p70, IL-6 and TNF-α. Hence, decreased production of these cytokines will reduce antibacterial inflammation and T_H_1 activation. In addition, upregulation of miR-99b in T_H_1 cells resulted in downregulation of TNF-α [131]. Therefore, these results suggest that miR-99b may decrease immune activation and increase the likelihood of MTB survival. The notion that MTB alters cellular miR expression was further enforced by the study from Kumar *et al*. [132], which revealed that MTB production of early secretory antigenic target 6 kD (ESAT-6) can reduce host miR expression. In mouse RAW264.7 cells, bone marrow-derived macrophages and human monocyte-derived macrophages, ESAT-6 downregulated Let-7f expression [132]. Let-7f downregulated A20, a negative regulator of NF-κB signaling, and the consequence of Let-7f downregulation was an increase in TRAF6 K63 deubiquitination by A20 and attenuated NF-κB signaling. Moreover, in the absence of A20, mouse macrophages expressed higher levels of IL-1β, IL-6, TNF, CCL2 and CCL3. This suggests that the bacterium could potentially benefit from decreased immune activation as well as decreased chemotaxis. Further analysis is required to determine whether these results can significantly improve pathogen survival *in vivo*. Moreover, while this study presents ESAT-6 as a likely cause for the Let-7f downregulation, it does not, however, present a mechanism by which ESAT6 is able to regulate expression of Let-7f.

In summary, bacteria may attenuate the immune response by inducing aberrant miR expression levels that interfere with the production of chemokines and cytokines. However, it is not always clear how these aberrations are induced and whether they are induced by the pathogen or that these alterations in miR expression are a means for the host to restrain excessive immune activation.

## 5. Concluding Remarks

The search for pathogen-derived miRs and the induction of specific host miRs has thus far indicated that some β and γ-herpesviridae indeed encode miRs, while HIV-1, EBV, KSHV, HCMV and various bacteria can induce the expression of specific host miRs. In general, the resulting effects of individual vmiRs or the induced host miRs are mild; however, the cumulative benefit of multiple miRs with pro-inflammatory targets and potential synergistic effects may be a valuable weapon in the existing immunocompromising pathogenic arsenal. What is more, vmiR knockdown experiments have frequently shown that diminished expression of specific vmiRs can reduce pathogen amplification. Although speculative, the results from these vmiR overexpression experiments do indicate the potential for one, or perhaps multiple, anti-miRs to ablate the pathogen-permissive environment during persistent or latent infections. Future elucidation of pathogen-specific use and abuse of miRs could contribute to the development of a miR-based approach for the treatment of specific pathogens. 

Most prominent findings:

- The combination of PAR-CLIP analysis and reporter assays provides clear indications that miR is functionally relevant.
- Evidence to support the claim that HIV-1 encodes functionally relevant vmiRs is limited.
- EBV, KSHV and HCMV-encoded miRs contribute, to varying degrees, to immune evasion and survival of the pathogen.
- Alterations in host cell miR expression during EBV, KSHV and HCMV infections may contribute to a cellular miR expression profile that promotes immune evasion and survival, thereby contributing to viral persistence.
- MTB infection affects expression of cellular miRs that decrease immune function, which may in some cases be pathogen specific and hence relevant for therapeutic targeting.
